# Effects of periodontal treatment on the medical status of patients with type 2 diabetes mellitus: a pilot study

**DOI:** 10.1186/s12903-017-0369-2

**Published:** 2017-04-21

**Authors:** Joichiro Hayashi, Akihiko Hasegawa, Kohei Hayashi, Takafumi Suzuki, Makiko Ishii, Hideharu Otsuka, Kazuhiro Yatabe, Seiichi Goto, Junichi Tatsumi, Kitetsu Shin

**Affiliations:** 1 Division of Periodontology, Department of Oral Biology and Tissue Engineering, Meikai University School of Dentistry, 1-1 Keyakidai, Sakado-shi, Saitama-ken 350-0283 Japan; 2 Meikai University School of Dentistry, Saitama-ken, Japan

**Keywords:** Type 2 diabetes mellitus, Periodontal treatment, Glycated hemoglobin, Urinary N-acetyl-β-D-glucosaminidase, γ-glutamyl transpeptidase

## Abstract

**Background:**

Studies have demonstrated that periodontal disease is associated with the development of systemic complications in patients with type 2 diabetes mellitus (T2DM). The purpose of this pilot study was to investigate which markers among various systemic disease parameters are affected by periodontal treatment in patients with T2DM.

**Methods:**

Twelve patients with T2DM were given oral hygiene instructions and subsequent subgingival scaling and root planing. The periodontal status was recorded, and blood and urine samples were taken to measure various parameters of glucose control and systemic status at baseline and 1 month following the periodontal treatment. Serum concentrations of tumor necrosis factor-α and high-sensitivity C-reactive protein were measured by enzyme-linked immunosorbent assay.

**Results:**

After the periodontal treatment, the glycated hemoglobin value was significantly improved. The levels of urinary N-acetyl-β-D-glucosaminidase and albumin, which are markers of renal dysfunction, also decreased significantly after treatment. Among the parameters measured in serum, the γ-glutamyl transpeptidase level, which is usually interpreted as a marker of liver dysfunction, was significantly reduced. The serum concentrations of tumor necrosis factor-α and high-sensitivity C-reactive protein were also significantly reduced by periodontal treatment.

**Conclusion:**

Within the limitations of this pilot study, periodontal treatment may be effective not only in improving metabolic control, but also in reducing the risk of diabetic kidney and liver disease in patients with T2DM.

## Background

Type 2 diabetes mellitus (T2DM) is a group of metabolic diseases characterized by hyperglycemia resulting from resistance to insulin action and an inadequate compensatory insulin secretory response. The chronic hyperglycemia of diabetes is associated with long-term damage, dysfunction, and failure of various organs.

Periodontitis, a common infectious disease, is regarded as a systemic inflammation. It produces some inflammatory cytokines in the local tissue and increases their circulating levels. Some studies have suggested a bidirectional relationship between T2DM and periodontitis [1]. It is known that diabetic patients have an increased risk of periodontitis, and that, once established, their periodontitis is more severe [2]. On the other hand, both direct and indirect evidence supports the concept that periodontal infection adversely affects glycemic control in people with diabetes [3].

Intervention studies examining the effects of periodontal treatment on glycemic control in diabetic patients with periodontal disease have generally shown a beneficial effect, with reduced glycated hemoglobin (HbA1c) levels [4–7], although not all studies confirmed this improvement [8]. Furthermore, some studies have demonstrated that periodontal disease is associated with the development of other systemic complications in diabetic patients, such as cardiovascular disease and renal disease [9–12]. However, it is still unclear whether periodontal treatment influences medical status other than glycemic control in such patients. The aim of this pilot study was to investigate which markers among various systemic disease parameters are affected by periodontal treatment in patients with T2DM.

## Methods

### Study subjects

Twelve subjects (mean age: 62.1 years; 7 males and 5 females) diagnosed with type 2 DM and chronic periodontitis were selected from the population referred to the Periodontal Clinic and to Internal Medicine at Meikai University Hospital. Criteria for inclusion were: type 2 DM diagnosed for >1 year, no change in DM treatment during the study, and presence of chronic periodontitis. Chronic periodontitis was diagnosed according to the criteria of the American Academy of Periodontology [13]. The exclusion criteria were: HbA1c ≥ 8%, any form of periodontal treatment in the previous 6 months, pregnancy, bleeding disorders, and any serious systemic diseases. Three subjects were taking prescribed insulin, and four subjects were smokers.

### Experimental design and treatment protocols

The patients received individually tailored intensive one-on-one oral hygiene instruction at several visits. Then, as the standard treatment for periodontitis, scaling and root planing per quadrant were performed using ultrasonic instruments and Gracey curettes (Hu-Friedy, Chicago, IL, USA). The periodontal parameters were recorded at baseline and at the first month following periodontal treatment. The following clinical parameters were recorded: number of teeth, probing depth (PD), and bleeding on probing (BOP). The PD and BOP were measured at six sites per tooth (mesio-buccal, mid-buccal, disto-buccal, disto-lingual, mid-lingual, and mesio-lingual). Venous blood and urine samples were taken at baseline and at the 1-month recall visit to monitor glucose control and systemic status. For the metabolic assessment, the blood samples were analyzed for fasting plasma glucose (FPG), immunoreactive insulin (IRI), and HbA1c (National Glycohemoglobin Standardization Program [14]) by a commercial laboratory (BML Inc., Tokyo, Japan). The homeostasis model assessment ratio (HOMA-R) index, which indicates the state of insulin resistance, was calculated based on the IRI and FPG [15]. The body mass index (BMI) was calculated by dividing the body weight by the square of the height. Concentrations of urinary N-acetyl-β-D-glucosaminidase (NAG) and albumin, which indicate renal disease such as diabetes-associated nephropathy, were measured in the urine samples by the commercial laboratory (BML Inc.).

### Biochemical parameters

Concentrations of total protein, albumin, bilirubin, aspartate aminotransferase (AST), alanine aminotransferase (ALT), alkaline phosphatase (ALP), lactate dehydrogenase (LDH), γ-glutamyl transpeptidase (GGT), urea nitrogen, creatinine, uric acid, IgG, IgM, IgA, IgD, low-density lipoprotein (LDL) cholesterol, high-density lipoprotein (HDL) cholesterol, and triglycerides in blood samples were measured by the commercial laboratory (BML Inc.).

### Measurement of tumor necrosis factor (TNF)-α and high-sensitivity C-reactive protein (hs-CRP)

The serum concentrations of TNF-α and hs-CRP were determined by enzyme-linked immunosorbent assay using commercially available kits for human TNF-α (Human TNF-α US, Invitrogen, Carlsbad, CA, USA) and human hs-CRP (C-Reactive Protein Kit, Hemagen Diagnostics, Waltham, MA, USA). All reagents and samples or standard solutions were added to a 96-well plate coated with murine monoclonal antibody against each protein and kept for 2 h. After subsequent treatment for 1–2 h with alkaline phosphatase-conjugated polyclonal antibody, reaction with *p*-nitrophenyl phosphate was carried out for 20 min. Trisodiumphosphate solution was then added to stop the reaction. The absorbance of each well was measured at 450 nm, and the concentration of each protein was calculated from standard curves.

### Statistical analysis

Statistical analyses were conducted using SPSS software (version 20.0; SPSS Japan, Tokyo, Japan). Differences between means at baseline and post-treatment were evaluated using the Wilcoxon signed-rank test. Spearman’s rank correlation coefficient was used to evaluate correlations between clinical parameters. Differences were considered significant at *P* ≤ 0.05.

## Results

Table 1 shows the values of clinical parameters before and after periodontal treatment. The mean PD, PD ≥ 4 mm, and BOP were significantly improved after non-surgical periodontal treatment. The periodontal treatment had no adverse effects.

**Table 1:** Periodontal parameters (mean ± SD) at baseline and post-treatment

	Baseline	Post-Treatment	*P* Value
Mean PD (mm)	4.26 ± 1.26	3.43 ± 0.90	0.005*
PD ≥ 4 mm (%)	48.1 ± 30.4	27.2 ± 26.0	0.005*
BOP (%)	49.4 ± 18.1	22.3 ± 19.8	0.005*

*Significant at *P* ≤ 0.05

The values of the systemic markers of T2DM are given in Table 2. The HbA1c level was significantly reduced after periodontal treatment. There was a tendency towards decreases in the levels of FPG, IRI, and HOMA-R, but these changes were not significant. There was little change in BMI. The levels of urinary NAG and albumin decreased significantly after treatment (Table 3).

**Table 2:** Diabetic parameters (mean ± SD) at baseline and post-treatment

	Baseline	Post-Treatment	*P* Value
FPG (mg/dl)	149.4 ± 55.9	117.2 ± 17.6	0.109
IRI (μU/ml)	6.5 ± 2.9	5.4 ± 1.9	0.110
HOMA-R	2.2 ± 1.0	1.4 ± 0.7	0.062
HbA1c (%)	7.2 ± 0.6	6.8 ± 0.6	0.033*
BMI (kg/m^2^)	24.5 ± 2.3	24.3 ± 1.9	0.838

*Significant at *P* ≤ 0.05

**Table 3:** Concentrations of NAG and albumin (mean ± SD) in urine at baseline and post-treatment

	Baseline	Post-Treatment	*P* Value
Urinary NAG (U/l)	8.1 ± 4.3	4.5 ± 2.1	0.005*
Urinary albumin (mg/l)	141.5 ± 266.4	51.7 ± 108.8	0.008*

*Significant at *P* ≤ 0.05

While the changes were not significant for most of the biochemical parameters in the blood after the non-surgical periodontal treatment, the reduction in the level of GGT was significant (Table 4). Although the change was not significant, periodontal treatment also tended to reduce the levels of AST, ALT, and LDH. The levels of urea nitrogen and creatinine tended to improve after treatment, but not significantly. The blood levels of total protein, albumin, bilirubin, and uric acid changed minimally after treatment. No significant changes were seen in immunoglobulins and lipid components after treatment. Although there was a slight decrease in the LDL cholesterol level and a slight increase in the HDL cholesterol level, the changes from baseline were not significant. Regarding the inflammatory biomarkers, the levels of TNF-α and hs-CRP were significantly decreased after periodontal treatment.

**Table 4:** Biochemical parameters (mean ± SD) in blood at baseline and post-treatment

	Baseline	Post-Treatment	*P* Value
Total protein (g/dl)	7.0 ± 0.7	7.2 ± 0.4	0.624
Albumin (g/dl)	4.3 ± 0.3	4.4 ± 0.3	0.552
Bilirubin (mg/dl)	0.7 ± 0.3	0.7 ± 0.3	0.681
AST (U/l)	23.3 ± 5.5	21.2 ± 4.0	0.165
ALT (U/l)	29.0 ± 11.8	23.2 ± 6.3	0.061
ALP (U/l)	163.0 ± 61.8	192.6 ± 62.3	0.051
LDH (U/l)	300 ± 113	215 ± 104	0.060
GGT (U/l)	33.4 ± 19.6	25.4 ± 9.7	0.025*
Urea nitrogen (mg/dl)	16.5 ± 4.5	14.8 ± 3.1	0.424
Creatinine (mg/dl)	0.90 ± 0.20	0.81 ± 0.10	0.053
Uric acid (mg/dl)	4.78 ± 0.90	4.81 ± 1.20	0.723
IgG (mg/dl)	1303 ± 290	1231 ± 223	0.919
IgM (mg/dl)	104 ± 38	109 ± 36	0.610
IgA (mg/dl)	337 ± 72	328 ± 88	0.859
IgD (mg/dl)	1.47 ± 1.51	1.71 ± 2.16	0.892
LDL cholesterol (mg/dl)	113.7 ± 24.0	108.2 ± 19.3	0.814
HDL cholesterol (mg/dl)	48.9 ± 10.0	51.8 ± 16.9	0.089
Triglycerides (mg/dl)	97.1 ± 51.3	103.7 ± 58.1	0.695
TNF-α (pg/ml)	4.89 ± 4.98	3.24 ± 2.43	0.026*
hs-CRP (μg/ml)	1.30 ± 3.33	1.21 ± 3.27	0.021*

*Significant at *P* ≤ 0.05

Table 5 presents the correlation analysis between clinical parameters that were significantly decreased after the periodontal treatment. Significant positive correlations were observed among mean PD, PD ≥ 4 mm, and BOP, and between urinary NAG and urinary albumin. In addition, PD ≥ 4 mm showed a significant positive correlation with urinary albumin. Urinary NAG and albumin were significantly correlated with hs-CRP. Correlations between reductions in clinical parameters are shown in Table 6. ∆Mean PD was significantly correlated with ∆PD ≥ 4 mm. Furthermore, ∆mean PD and ∆PD ≥ 4 mm showed significant positive correlations with ∆TNF-α.

**Table 5:** Correlation (r) values between clinical parameters at baseline

	Mean PD	PD ≥4 mm	BOP	HbA1c	Urinary NAG	Urinary albumin	GGT	TNF-α
PD ≥ 4 mm	0.878*							
BOP	0.771*	0.608*						
HbA1c	−0.152	−0.325	0.138					
Urinary NAG	0.464	0.501	0.389	−0.24				
Urinary albumin	0.643	0.690*	0.631	−0.109	0.726*			
GGT	−0.024	0.021	−0.192	−0.376	0.148	0.176		
TNF-α	0.379	0.393	0.021	−0.210	0.359	−0.052	0.243	
hs-CRP	0.181	0.206	0.255	−0.010	0.663*	0.827*	0.174	−0.001

*Significant at *P* ≤ 0.05

**Table 6:** Correlation (r) values between reductions in clinical parameters

	∆Mean PD	∆PD ≥4 mm	∆BOP	∆HbA1c	∆Urinary NAG	∆Urinary albumin	∆GGT	∆TNF-α
∆PD ≥ 4 mm	0.730*							
∆BOP	0.351	0.289						
∆HbA1c	−0.019	−0.064	0.456					
∆Urinary NAG	0.522	0.24	0.224	−0.313				
∆Urinary albumin	0.328	0.178	0.134	−0.142	0.599			
∆GGT	−0.508	−0.317	−0.246	−0.506	−0.116	0.108		
∆TNF-α	0.677*	0.621*	0.309	−0.223	0.407	−0.146	−0.012	
∆hs-CRP	0.081	−0.007	−0.054	0.136	0.341	0.403	−0.212	−0.018

*Significant at *P* ≤ 0.05

## Discussion

In the present study of T2DM patients, urinary NAG and albumin, which are markers of diabetic renal disease, decreased significantly after periodontal treatment. The serum levels of urea nitrogen and creatinine, which are markers of renal damage, also tended to improve after treatment. In addition, the concentration of urinary albumin was significantly correlated with PD ≥4 mm at baseline. Therefore, it was suggested that periodontal treatment in patients with T2DM might improve kidney function, directly or indirectly. A proposed mechanism for the effect of periodontitis on the development of renal disease is as follows. Individuals with significant periodontitis have chronic and recurrent episodes of low level bacteremia [16, 17]. Circulating periodontal pathogens in the bloodstream could lead to renal endothelial damage. The lipopolysaccharide of periodontal pathogens may induce the secretion of inflammatory mediators, including interleukin 6, TNF-α, prostaglandin E2, and thromboxane B2. These cytokines accelerate atherogenesis, thrombus formation, and platelet aggregation [18]. Additionally, thromboxane has potent vasoconstrictive properties [19], and, speculatively, chronic production of this substance may lead to a chronic decrease in renal blood flow [12]. On the other hand, periodontal treatment may affect renal function indirectly. Diabetic renal disease is one of the diabetic complications. As a result of improved glycemic control by periodontal treatment, renal function may improve.

Although increased serum GGT activity is usually interpreted as a marker of alcohol abuse and liver dysfunction, parallel evidence from epidemiological studies suggests that higher serum GGT levels are also associated with the metabolic syndrome [20], hypertension [21], and diabetes [22, 23]. In the present study, the GGT level decreased after periodontal treatment. The levels of AST and ALT, which are also markers of liver damage, tended to decrease. Regarding periodontal diseases, there are few reports about GGT. It has been reported that GGT blood levels were positively associated with attachment level in alcoholic persons, but not in non-alcoholic persons [24]. On the other hand, there are some reports about a relationship between periodontal diseases and liver disease [25, 26]. The present data appear to support such reports. There are two possible pathways for liver injury by periodontal disease, a direct pathway by periodontopathic bacteria and an indirect pathway by cytokines including TNF-α induced by bacteria and lipopolysaccharides. TNF-α plays a role in the pathogenesis of liver injury [27]. In the present study, GGT might have improved due to blockage of these two pathways by periodontal treatment.

The good healing response of T2DM patients to non-surgical therapy in the present study confirms the results of previous investigations [28, 29]. The HbA1c level was significantly decreased after non-surgical periodontal treatment. The possible confounding factors may include changes in DM treatment and BMI during the study. However, since no changes were observed in them, this suggests that glycemic control might have been improved by periodontal therapy. Although periodontal therapy was performed for patients with T2DM whose HbA1c was > 10% in some studies, HbA1c levels should be controlled before periodontal therapy to avoid the risk of postoperative infection [30]. In this study, the HbA1c level was controlled to less than 8% to adapt to the clinical situation. The mean HbA1c at baseline was 7.2%, which is lower than that in other studies. Nevertheless, it was noted that the diabetic parameters were significantly decreased.

In periodontitis, up-regulated proinflammatory cytokines contribute to periodontal tissue destruction [31]. It has been demonstrated that TNF-α has a role in osteoclastogenesis in mice [32], and recombinant TNF-α has been shown to accelerate periodontitis in rats [33]. TNF-α also induces insulin resistance in T2DM [34]. In addition, it has been shown that urinary TNF-α levels are associated with the presence and severity of microalbuminuria in patients with T2DM [35]. Thus, TNF-α appears to be a key mediator in the pathogenesis of periodontal disease, T2DM, and diabetic renal disease. In patients with periodontitis and T2DM, increased TNF-α levels are associated with increased attachment loss [36]. In the present study, serum TNF-α was shown to be significantly decreased by periodontal treatment. Furthermore, ∆mean PD and ∆PD ≥ 4 mm were significantly correlated with ∆TNF-α. Therefore, it was suggested that periodontal treatment is effective in improving metabolic control in patients with T2DM, possibly through reduction in the serum concentration of TNF-α and decreased insulin resistance. Though some studies have shown improved glycemic control with periodontal treatment in T2DM patients through reduction of peripheral TNF-α concentrations [37, 38], no reduction in serum TNF-α levels has been found following periodontal therapy in T2DM patients in other studies [39, 40]. However, a recent systematic review found that periodontal treatment reduced serum levels of TNF-α and CRP in T2DM patients [41].

CRP, an important mediator of inflammation, is mainly synthesized in the liver. Elevated serum CRP is associated with both periodontitis [42, 43] and T2DM [44]. Meta-analyses and systematic reviews of the relationship of CRP to periodontitis have shown consistently higher CRP levels in patients than in controls [42, 43]. In a meta-analysis of 4 randomized clinical trials [43], CRP levels were significantly reduced after the start of periodontal treatment. There is agreement between the findings of both meta-analyses and the results of the present study. In addition, the hs-CRP level was shown to be correlated with urinary NAG and albumin in the present study. A recent study showed that serum hs-CRP levels were associated with a subsequent risk of developing diabetic nephropathy in type 2 diabetic patients [45].

## Conclusion

This pilot study showed that clinically successful non-surgical periodontal treatment improved not only the level of glycemic control, but also the levels of NAG and albumin in the urine and GGT in the serum among various systemic disease markers. These findings suggest that periodontal treatment is effective not only in improving metabolic control, but also in reducing the risk of diabetic renal and liver disease in patients with T2DM. However, the present study has one limitation in that it lacked a group of T2DM patients who were not undergoing periodontal treatment; thus, how these patients would progress remains unknown. Another limitation was the small sample size. Thus, future studies of T2DM patients involving larger samples and a control group are needed to clarify how metabolic control and markers of diabetic liver and kidney diseases are affected by periodontal therapy.

## Abbreviations

ALP: Alkaline phosphatase
ALT: Aminotransferase
AST: Aspartate aminotransferase
BMI: Body mass index
BOP: Bleeding on probing
FPG: Fasting plasma glucose
GGT: γ-glutamyl transpeptidase
HbA1c: Glycated hemoglobin
HDL: High-density lipoprotein
HOMA-R: Homeostasis model assessment ratio
hs-CRP: High-sensitivity C-reactive protein
IRI: Immunoreactive insulin
LDH: Lactate dehydrogenase
LDL: Low-density lipoprotein
NAG: N-acetyl-β-D-glucosaminidase
PD: Probing depth
T2DM: Type 2 diabetes mellitus
TNF: Tumor necrosis factor
